# Supplementary material for: Systematic review of exercise for the treatment of pediatric metabolic dysfunction-associated steatotic liver disease
Source: PLoS One. 2024 Dec 10;19(12):e0314542. doi: 10.1371/journal.pone.0314542 (PMC11630624; doi:10.1371/journal.pone.0314542)
Supplement: S1 File — (DOCX) [file pone.0314542.s001.docx]

**Review Protocol**

**Title**

Systematic review of exercise for the treatment of pediatric metabolic dysfunction-associated steatotic liver disease

**Review Question**

What are the effects of exercise on liver disease in children with NAFLD and/or MASLD?

**Search Strategy**

A literature search will be conducted in Cochrane Central Register of Controlled Trials in the Cochrane Library, PubMed, Embase, Web of Science Core Collection, and CINAHL Complete on EBSCOhost, and Google Scholar from the date of inception by two independent investigators. The search strategy was developed with a search specialist, based on medical subject subheadings (MeSH) terms and keywords. Reference lists of all primary studies and relevant review articles will also be searched. No date or country restrictions will be applied.

See detailed electronic search strategy on pages 5-7.

**Types of study to be included**

Full-text English language publications of interventional studies including RCTs, NRCTs, and uncontrolled trials published in peer-reviewed journals will be included.

**Condition or domain being studied**

Exercise intervention in children with NAFLD or hepatic steatosis associated with overweight or obesity

**Participants/population**

Children and adolescents (aged 19 years or younger) diagnosed with steatotic liver disease or at risk for steatotic liver disease due to overweight or obesity

**Intervention(s), exposure(s)**

All types of exercise intervention will be considered. They can occur in any setting and be supervised or unsupervised. Interventions that include nutrition counseling, psychological counseling, and motivational interviewing will be considered if administered equally to the comparison groups.

**Comparator(s)/control**

We will consider the following comparisons:

- Exercise versus no exercise;
- Exercise plus lifestyle intervention versus lifestyle intervention alone;
- One type of exercise versus another type of exercise;
- One type of exercise plus lifestyle intervention versus another type of exercise plus lifestyle intervention.

We will also include uncontrolled studies with exercise-only interventions.

**Context**All studies must report on outcomes relevant to our review objectives.

**Main outcome(s)**

Three outcomes will be evaluated:

1. Quantitative imaging assessment of hepatic steatosis
2. Liver chemistries (ALT and GGT)
3. Changes in liver histology

**Measures of effect**

Varies by outcome

**Additional outcome(s)**

None

**Data extraction (selection and coding)**

Titles and abstracts of studies identified using our search strategy will be independently reviewed by two reviewers against our predefined inclusion and exclusion criteria. Discrepancies will be resolved through discussion and arbitration by a third reviewer. Where multiple papers report results from the same study they will be grouped for further assessment. Full text assessment will also be performed independently by two reviewers with any discrepancies resolved through discussion or consultation with a third reviewer. A standardized pre-piloted form will be used to extract data from the included studies for assessment of study quality and synthesis. Extracted information will include:

- General information: Study title, study ID, author(s), year of publication, journal/source, and country of origin.
- Study characteristics: Diseases studied, study design, study date, duration of participation, and setting.
- Participant characteristics: Description of the study population, inclusion and exclusion criteria, method of recruitment, sample size, clusters (if applicable), method of randomization, baseline imbalances between arms, withdraws and exclusions, age rage, mean age, sex, race/ethnicity, height, weight, BMI, liver fat, ALT, AST, GGT, diagnoses, and comorbidities.
- Intervention details: Number of participants randomized to each group, detailed description of the exercise type, frequency, intensity, duration of each session, duration of the treatment period, co-interventions, integrity of delivery (i.e., prescribed or supervised), and compliance.
- Outcome information: Data on both primary and secondary outcomes, time points measured, assessment method, and statistical methods used.
- Additional information: Key conclusions of study authors.

**Risk of bias (quality) assessment**

Risk of bias will be assessed using the ROB2 tool for randomized controlled trials and ROBINS tool for NRCTs and uncontrolled studies.

**Strategy for data synthesis**

A descriptive synthesis of the findings from the included studies organized around type of intervention, study population, and outcomes will be performed. All studies will be included in tables that present the baseline participant characteristics, intervention descriptions, and results.

**Contact details for further information**

Martha Smith

[martha.smith@my.rfums.org](mailto:martha.smith@my.rfums.org)

**Organizational affiliation of the review**

University of California San Diego School of Medicine

**Review team members and their organizational affiliations**

Martha R Smith^1^

Jeffrey B Schwimmer^2,3^

Elizabeth L Yu^2,3^

Ghattas J Malki^2^

Nidhi P Goyal^2,3^

Kimberly P Newton^2,3^

Karen M Heskett^4^

^1^Chicago Medical School, Rosalind Franklin University of Medicine and Science, North Chicago, Illinois, United States of America

^2^Department of Pediatrics, Division of Gastroenterology, Hepatology, and Nutrition, University of California San Diego School of Medicine, La Jolla, California, United States of America

^3^Department of Pediatrics, Division of Gastroenterology, Rady Children’s Hospital, San Diego, California, United States of America

^4^The Library, University of California San Diego, La Jolla, California, United States of America

**Type and method of review**

Narrative synthesis, systematic review

**Anticipated or actual start date**

23 May 2023

**Anticipated completion date**

01 March 2024

**Funding sources/sponsors**

None

**Conflicts of interest**

None

**Country**

United States of America

**Stage of review**

Review ongoing

**Registration**

This protocol was not registered

**Electronic search strategy**

| Database | Time span | Search strategy |
| --- | --- | --- |
| Cochrane Central Register of Controlled Trials (CENTRAL) in the Cochrane Library | 2023, Issue 6 | #1 MeSH descriptor: [non-alcoholic fatty liver disease] explode all trees  #2 MeSH descriptor: [fatty liver] explode all trees  #3 non-alcoholic fatty liver disease OR nonalcoholic steatohepatitis OR nonalcoholic fatty liver disease OR nafld OR nonalcoholic fatty liver  #4 MeSH descriptor: [exercise] explode all trees  #5 MeSH descriptor: [exercise therapy] explode all trees  #6 exercise therap* OR exercise* OR physical activit*  #7 MeSH descriptor: [adolescent] explode all trees  #8 MeSH descriptor: [child] explode all trees  #9 MeSH descriptor: [infant] explode all trees  #10 MeSH descriptor: [pediatrics] explode all trees  #11 infant* OR pediatric* OR child* OR adolescent*  #12 #1 or #2 or #3  #13 #4 or #5 or #6  #14 #7 or #8 or #9 or #10 or #11  #15 #12 and #13 and #14 |
| PubMed (PubMed.gov) | 1946 to 5 June 2023 | 1. "adolescent"[MeSH] or "child"[MeSH] or "infant"[MeSH] or child*[tiab] or adolescent*[tiab] or infant*[tiab] or "pediatrics"[MeSH] or pediatric*[tiab] 2. "exercise"[MeSH] or exercise*[tiab] or physical activit*[tiab] or "exercise therapy"[Mesh] or exercise therap*[tiab] 3. "non-alcoholic fatty liver disease"[MeSH] or "fatty liver"[MeSH] or non-alcoholic fatty liver disease[tiab] or nonalcoholic steatohepatitis[tiab] or nonalcoholic fatty liver disease[tiab] 4. 1 and 2 and 3 |
| Embase (Embase.org) | 1974 to 5 June 2023 | 1. 'juvenile'/exp or 'pediatrics'/exp or child*:ti,ab,kw or adolescent*:ti,ab,kw or Infant*:ti,ab,kw or pediatric*:ti,ab,kw 2. 'physical activity'/exp or 'exercise'/exp or exercise*:ti,ab,kw or ‘physical activit*’:ti,ab,kw or ‘exercise therap*’:ti,ab,kw 3. 'nonalcoholic fatty liver'/syn or ‘non-alcoholic fatty liver disease’:ti,ab,kw or steatohepatitis:ti,ab,kw or ‘nonalcoholic steatohepatitis’:ti,ab,kw or ‘nonalcoholic fatty liver disease’:ti,ab,kw or NAFLD:ti,ab,kw 4. 1 and 2 and 3 |
| Web of Science Core Collection | 1900 to 6 June 2023 | #9: #4 OR #8  #8: #5 AND #6 AND #7  #7: TI=("nonalcoholic fatty liver disease" or nafld or "nonalcoholic steatohepatitis" or "non-alcoholic fatty liver disease" or "non alcoholic fatty liver disease")  #6: TI=(child* or pediatric* or infant* or adolescent*)  #5: TI=(exercise* or "exercise therap*" or "physical activit*")  #4: #1 AND #2 AND #3  #3: AB=("nonalcoholic fatty liver disease" or nafld or "nonalcoholic steatohepatitis" or "non-alcoholic fatty liver disease" or "non alcoholic fatty liver disease")  #2: AB=(child* or pediatric* or infant* or adolescent*)  #1: AB=(exercise* or "exercise therap*" or "physical activit*") |
| CINAHL Complete on EBSCOhost | 1982 to 5 June 2023 | S1 TI (nonalcoholic fatty liver disease or nafld or nonalcoholic steatohepatitis or (MH "Fatty Liver+") or (MH "Nonalcoholic Fatty Liver Disease")) OR AB (nonalcoholic fatty liver disease or nafld or nonalcoholic steatohepatitis or (MH "Fatty Liver+") or (MH "Nonalcoholic Fatty Liver Disease"))  S2 TI (exercise* or physical activit* or exercise therap* or (MH "Therapeutic Exercise+") or (MH "Exercise+")) OR AB (exercise* or physical activit* or exercise therap* or (MH "Therapeutic Exercise+") or (MH "Exercise+"))  S3 TI (pediatric* or child* or infant* or adolescent* or (MH "Child+") or (MH "Adolescence+") or (MH "Pediatrics+")) OR AB ( pediatric* or child* or infant* or adolescent* or (MH "Child+") or (MH "Adolescence+") or (MH "Pediatrics+"))  S4 S1 AND S2 AND S3 |
| Google Scholar |  | Keyword search: nonalcoholic fatty liver disease OR NAFLD OR nonalcoholic steatohepatitis OR non-alcoholic fatty liver disease OR non alcoholic fatty liver disease; child OR pediatric OR infant OR adolescent; exercise OR exercise therapy OR physical activity OR physical activities. |
