# Supplementary material for: Systematic review of exercise for the treatment of pediatric metabolic dysfunction-associated steatotic liver disease
Source: PLoS One. 2024 Dec 10;19(12):e0314542. doi: 10.1371/journal.pone.0314542 (PMC11630624; doi:10.1371/journal.pone.0314542)
Supplement: S2 File — (DOCX) [file pone.0314542.s002.docx]

| Database | Time span | Search strategy |
| --- | --- | --- |
| Cochrane Central Register of Controlled Trials (CENTRAL) in the Cochrane Library | 2023, Issue 6 | #1 MeSH descriptor: [non-alcoholic fatty liver disease] explode all trees  #2 MeSH descriptor: [fatty liver] explode all trees  #3 non-alcoholic fatty liver disease OR nonalcoholic steatohepatitis OR nonalcoholic fatty liver disease OR nafld OR nonalcoholic fatty liver OR masld^+^ OR metabolic dysfunction-associated steatotic liver disease^+^  #4 MeSH descriptor: [exercise] explode all trees  #5 MeSH descriptor: [exercise therapy] explode all trees  #6 exercise therap* OR exercise* OR physical activit*  #7 MeSH descriptor: [adolescent] explode all trees  #8 MeSH descriptor: [child] explode all trees  #9 MeSH descriptor: [infant] explode all trees  #10 MeSH descriptor: [pediatrics] explode all trees  #11 infant* OR pediatric* OR child* OR adolescent*  #12 #1 or #2 or #3  #13 #4 or #5 or #6  #14 #7 or #8 or #9 or #10 or #11  #15 #12 and #13 and #14 |
| PubMed (PubMed.gov) | 1946 to 5 June 2023 | 1. "adolescent"[MeSH] or "child"[MeSH] or "infant"[MeSH] or child*[tiab] or adolescent*[tiab] or infant*[tiab] or "pediatrics"[MeSH] or pediatric*[tiab] 2. "exercise"[MeSH] or exercise*[tiab] or physical activit*[tiab] or "exercise therapy"[Mesh] or exercise therap*[tiab] 3. "non-alcoholic fatty liver disease"[MeSH] or "fatty liver"[MeSH] or non-alcoholic fatty liver disease[tiab] or nonalcoholic steatohepatitis[tiab] or nonalcoholic fatty liver disease[tiab] or masld[tiab]^+^ or metabolic dysfunction-associated steatotic liver disease[tiab]^+^ 4. 1 and 2 and 3 |
| Embase (Embase.org) | 1974 to 5 June 2023 | 1. 'juvenile'/exp or 'pediatrics'/exp or child*:ti,ab,kw or adolescent*:ti,ab,kw or Infant*:ti,ab,kw or pediatric*:ti,ab,kw 2. 'physical activity'/exp or 'exercise'/exp or exercise*:ti,ab,kw or ‘physical activit*’:ti,ab,kw or ‘exercise therap*’:ti,ab,kw 3. 'nonalcoholic fatty liver'/syn or ‘non-alcoholic fatty liver disease’:ti,ab,kw or steatohepatitis:ti,ab,kw or ‘nonalcoholic steatohepatitis’:ti,ab,kw or ‘nonalcoholic fatty liver disease’:ti,ab,kw or NAFLD:ti,ab,kw or MASLD:ti,ab,kw^+^ or ‘metabolic dysfunction-associated steatotic liver disease’:ti,ab,kw^+^ 4. 1 and 2 and 3 |
| Web of Science Core Collection | 1900 to 6 June 2023 | #9: #4 OR #8  #8: #5 AND #6 AND #7  #7: TI=("nonalcoholic fatty liver disease" or nafld or "nonalcoholic steatohepatitis" or "non-alcoholic fatty liver disease" or "non alcoholic fatty liver disease" or masld^+^ or “metabolic dysfunction-associated steatotic liver disease”^+^)  #6: TI=(child* or pediatric* or infant* or adolescent*)  #5: TI=(exercise* or "exercise therap*" or "physical activit*")  #4: #1 AND #2 AND #3  #3: AB=("nonalcoholic fatty liver disease" or nafld or "nonalcoholic steatohepatitis" or "non-alcoholic fatty liver disease" or "non alcoholic fatty liver disease" or masld^+^ or “metabolic dysfunction-associated steatotic liver disease”^+^)  #2: AB=(child* or pediatric* or infant* or adolescent*)  #1: AB=(exercise* or "exercise therap*" or "physical activit*") |
| CINAHL Complete on EBSCOhost | 1982 to 5 June 2023 | S1 TI (masld^+^ or metabolic dysfunction-associated steatotic liver disease^+^ or nonalcoholic fatty liver disease or nafld or nonalcoholic steatohepatitis or (MH "Fatty Liver+") or (MH "Nonalcoholic Fatty Liver Disease")) OR AB (masld^+^ or metabolic dysfunction-associated steatotic liver disease^+^ or nonalcoholic fatty liver disease or nafld or nonalcoholic steatohepatitis or (MH "Fatty Liver+") or (MH "Nonalcoholic Fatty Liver Disease"))  S2 TI (exercise* or physical activit* or exercise therap* or (MH "Therapeutic Exercise+") or (MH "Exercise+")) OR AB (exercise* or physical activit* or exercise therap* or (MH "Therapeutic Exercise+") or (MH "Exercise+"))  S3 TI (pediatric* or child* or infant* or adolescent* or (MH "Child+") or (MH "Adolescence+") or (MH "Pediatrics+")) OR AB ( pediatric* or child* or infant* or adolescent* or (MH "Child+") or (MH "Adolescence+") or (MH "Pediatrics+"))  S4 S1 AND S2 AND S3 |
| Google Scholar |  | Keyword search: MASLD^+^\|“metabolic dysfunction-associated steatotic liver disease”^+^\|“nonalcoholic fatty liver disease”\|NAFLD\|“nonalcoholic steatohepatitis”\|“non-alcoholic fatty liver disease”; exercise\|“exercise therapy”\|“physical activity”; child\|pediatric\|adolescent\|infant. |

^+^Keyword term used in the updated search performed on August 8, 2024 to reflect the new terminology for steatotic liver disease.
