# Supplementary material for: Systematic review of exercise for the treatment of pediatric metabolic dysfunction-associated steatotic liver disease
Source: PLoS One. 2024 Dec 10;19(12):e0314542. doi: 10.1371/journal.pone.0314542 (PMC11630624; doi:10.1371/journal.pone.0314542)
Supplement: S3 File — (DOCX) [file pone.0314542.s003.docx]

**Detailed methods for assessing risk of bias in included studies**

For randomized trials, we evaluated the following domains:

- Bias arising from the randomization process
- Bias due to deviations from intended interventions
- Bias due to missing outcome data
- Bias in measurement of the outcome
- Bias in selection of the reported result

We categorized the studies as having low, high, or some concerns for risk of bias in each assessed domain. An overall low risk of bias was assigned when all domains were assessed as low risk, some concerns for overall risk of bias when at least one domain raised some concerns without any domain being at high risk, and high risk of bias when at least one domain was assessed as high risk or if multiple domains raised concerns that substantially lowered confidence in the result.

For non-randomized studies of interventions, we evaluated the following domains:

- Bias due to confounding
- Bias in selection of participants into the study
- Bias in classification of interventions
- Bias due to deviations from intended interventions
- Bias due to missing data
- Bias in measurement of outcomes
- Bias in selection of the reported result

These studies were categorized as having low, moderate, serious, or critical risk of bias, or no information for each assessed domain. An overall low risk of bias was assigned when all domains were assessed as low risk, moderate overall risk of bias when all domains were assessed as low or moderate risk, serious overall risk of bias when at least one domain was assessed as high risk without any domain at critical risk, and critical overall risk of bias when at least one domain was assessed as at critical risk.
