# Supplementary material for: Systematic review of exercise for the treatment of pediatric metabolic dysfunction-associated steatotic liver disease
Source: PLoS One. 2024 Dec 10;19(12):e0314542. doi: 10.1371/journal.pone.0314542 (PMC11630624; doi:10.1371/journal.pone.0314542)
Supplement: S4 File — (DOCX) [file pone.0314542.s004.docx]

| **Study** | **Type of study** | **Length**  **(weeks)** | **Exercise Intervention Arms** | **Exercise Frequency** | **Exercise Intervention Details** | **Dietary or Other Modifications** |
| --- | --- | --- | --- | --- | --- | --- |
| Lee, S. et al. 2012 | RCT | 12 | Aerobic exercise | 3x/wk for 60 min each; supervised | Weeks 1-2: 40 min at 50% VO2 peak  Weeks 3-12: 60 min at 60-75% VO2 peak | Weight maintenance diet:  - 55% carbs  - 15-20% protein  - 20-25% fat |
|  |  |  | Resistance exercise | 3x/wk for 60 min each; supervised | 10 whole-body exercises  Weeks 1-4: 1-2 sets of 8-12 reps at 60% of one-rep max  Weeks 4-12: 2 sets of 8-12 reps to fatigue |  |
|  |  |  | Non-exercise control | N/A | No structured physical activity |  |
| Iraji et al. 2021 | NRCT | 8 | School-based exercise | 3x/wk; supervised | Weeks 1-2: 50 min of 20m shuttle run test, futsal games, jump rope. ~300-350 kcal consumed  Weeks 3-5: 55 min of 20m shuttle run test, basketball, jump rope. ~350-400 kcal consumed  Weeks 6-8: 60 min of 20m shuttle run test, handball games, jump rope. ~400-450 kcal consumed | None |
|  |  |  | High-intensity interval training | 3x/wk; supervised | Weeks 1-2: Two sets of 6 reps consisting of running at max aerobic speed for 30s followed by 30s active recovery. Four-minute rest between sets. ~250-300 kcal consumed  Weeks 3-5: Two sets of 7 reps consisting of running at max aerobic speed for 30s followed by 30s active recovery. Four-minute rest between sets. ~300-350 kcal consumed  Weeks 6-8: Two sets of 8 reps consisting of running at max aerobic speed for 30s followed by 30s active recovery. Four-minute rest between sets. ~250-300 kcal consumed |  |
| Lee, S. et al. 2013 | RCT | 12 | Aerobic exercise | 3x/wk for 60 min each; supervised | Weeks 1-2: 40 min at 50% VO2 peak  Weeks 3-12: 60 min at 60-75% VO2 peak | Weight maintenance diet:  - 55% carbs  - 15-20% protein  - 20-25% fat |
|  |  |  | Resistance exercise | 3x/wk for 60 min each; supervised | 10 whole-body exercises  Weeks 1-4: 1-2 of 8-12 reps at 60% of one-rep max  Weeks 4-12: 2 sets of 8-12 reps to fatigue |  |
|  |  |  | Non-exercise control | N/A | No structured physical activity |  |
| Davis et al. 2011 | RCT | 16 | Circuit training | 2x/wk for 60-90 min; supervised | Weeks1-4: 8-9 circuits with two strength training (1 min each) and one cardiovascular exercise (2 min). 70-75% HRmax  Weeks 5-10: 6-7 circuits repeated twice with two strength training (1 min each) and one cardiovascular exercise (2.5 min). 70-80% HRmax  Weeks 11-16: 7-8 circuits repeated twice with two strength training (1 min each) and one cardiovascular exercise (3 min). 70-85% HRmax | None |
|  |  |  | Circuit training + motivational interviewing | 2x/wk for 60-90min; supervised | Weeks 1-4: 8-9 circuits with two strength training (1 min each) and one cardiovascular exercise (2 min). 70-75% HRmax  Weeks 5-10: 6-7 circuits repeated twice with two strength training (1 min each) and one cardiovascular exercise (2.5 min). 70-80% HRmax  Weeks 11-16: 7-8 circuits repeated twice with two strength training (1 min each) and one cardiovascular exercise (3 min). 70-85% HRmax | 4 individual + 4 group motivational interviewing sessions |
|  |  |  | Control | N/A | No intervention | No intervention |
| Lee, YH. et al. 2010 | RCT | 10 | Aerobic exercise | 3x/wk for 60 min (2x supervised, 1x unsupervised) | Exercise based on child interest (soccer, hockey, jump rope, etc). Adjusted to VO2 max 60-80%, HRmax 70-90%, 300-400 kcal per session | None |
|  |  |  | Combined exercise | 3x/wk for 60 min (2x supervised, 1x unsupervised) | Two circuit weight training routines + one aerobic exercise routine each session  Circuit consisted of 8-10 aerobic and resistance training exercises for 30s each with 10 sec rest. Resistance intensity was 70-80% max strength |  |
|  |  |  | Control | N/A | Maintained former lifestyle |  |
| Hay et al. 2016 | RCT | 24 | High-intensity interval exercise | 3x/wk for 30-45 min; supervised | Self-selected exercise to reach 70-85% heart rate reserve  Duration adjusted for ~350 kcal consumed per session | None |
|  |  |  | Moderate-intensity continuous exercise | 3x/wk for 30-45 min; supervised | Self-selected exercise to reach 40-55% heart rate reserve  Duration adjusted for ~350 kcal consumed per session |  |
|  |  |  | Control | N/A | Maintained former lifestyle |  |
| Tas et al. 2023 | RCT | 4 | High-intensity interval training | 3x/wk for 45 min; supervised | 5- to 10-min low-intensity warm-up, 10 1-min intervals using exercise equipment (elliptical machine, bike, or treadmill) at a work rate that elicited 80% to 90% of the maximal heart rate determined during a VO2 peak test with 2 min of walking between each effort, 5- to 10-min cool-down at a low intensity | None |
|  |  |  | Control | N/A | Maintained former lifestyle |  |
| Van der Heijden et al. 2010(a) | Uncontrolled | 12 | Aerobic exercise | 4x/wk (2x supervised, 2x unsupervised) | >70% VO2 peak through aerobic exercise on treadmill, elliptical, or bicycle based on preference | None except participants received an identical 7-day low carbohydrate/high fat diet before baseline and postexercise measurements to exclude the effects of dietary intake |
| Van der Heijden et al. 2010(b) | Uncontrolled | 12 | Resistance exercise | 2x/wk for 1hr; supervised | Full body straight training (chest, back, triceps, biceps, shoulders, quads, hamstrings, calves, gluts, and abs)  Weeks 1-2: 2-3 sets of 8-12 reps with weight set to ~50% of 3 rep max  Gradual increase in weight/reps  Weeks 9-12: 3 sets of 15-20 reps with weight set to ~85% of 3 rep max | None except participants received an identical 7-day low carbohydrate/high fat diet before baseline and postexercise measurements to exclude the effects of dietary intake |
| Bell et al. 2007 | Uncontrolled | 8 | Circuit training | 3x/wk for 1hr; supervised | Weeks 1-2: alternating between 1 min of cycling at 65% HRmax and 1min of 12 reps of weight lifting at 55% pretraining max contraction  Weeks 3-8: alternating between 1 min of cycling at 85% HRmax and 1min of 12 reps of weight lifting at 65% pretraining max contraction | None |
| Labayen et al. 2020 | NRCT | 22 | Lifestyle, psycho-educational intervention | N/A | No intervention | 45 min lifestyle program every 2wk weeks  45 min psychoeducation program every 2wk |
|  |  |  | Exercise with lifestyle, psycho-educational intervention | 3x/wk for 90min; supervised | Included 60min game-based cardiovascular endurance training and 10min muscle strength exercises  No set goal for exercise intensity; high-intensity exercise was maintained for 49% of the time and moderate-intensity for 32% |  |
| Hasson et al. 2012 | RCT | 16 | Strength training + nutrition education | 2x/wk for 60min; supervised | Per week, one session focused on compound lower-body exercises and isolated upper-body exercises; other session focused on compounded upper-body exercises and isolated lower-body exercises  Progressively increased number of sets and resistance | Motivational interviewing session monthly  Nutrition education 1x/wk for 90 min with goals of (1) ≤10% total daily calorie intake from added sugars, (2) consuming >14g/1000kcal dietary fiber daily |
|  |  |  | Nutrition education | N/A | No intervention |  |
|  |  |  | Control | N/A | No intervention | No intervention |
| Lee, S. et al. 2019 | RCT | 24 | Aerobic exercise | 3x/wk for 60 min; supervised | Treadmill or elliptical moderate-intensity with 50-65% VO2 peak | Follow target calorie intake to ensure negative energy balance was induced by regular exercise alone and not from dieting |
|  |  |  | Resistance exercise | 3x/wk for 60 min; supervised | Two sets of 8 exercises (12-15 reps) using weight machines targeting major muscle groups |  |
|  |  |  | Combined aerobic and resistance exercise | 3x/wk for 60 min; supervised | 30 min of treadmill or elliptical with 50-65% VO2 peak and 30min of resistance exercises consisting of one set of 8 exercises (12-15 reps) using weight machines targeting major muscle groups |  |
| González-Ruíz et al. 2022 | RCT | 24 | High-intensity training plus physical education (HIPE) | 3x/wk for 1hr; supervised | 75-85% HRmax with goal consumption of 300-500 kcal per session  Play games previously evaluated in other studies | Participants reviewed a hygiene-dietary management plan that included educational and nutritional support throughout study |
|  |  |  | Low-to-moderate intensity training plus physical education (LIPE) | 3x/wk for 1hr; supervised | 55-75% HRmax with goal consumption of 300-500 kcal per session  Play games previously evaluated in other studies |  |
|  |  |  | Combined HIPE and LIPE | 3x/wk for 1hr; supervised | 30 min aerobic training and 30 min resistance training that involved training from both HIPE and LIPE. Play games from HIPE and LIPE groups |  |
|  |  |  | Standard physical education lessons | 60 min/wk; supervised | Low-to-moderate intensity physical education |  |
| De Piano et al. 2012 | RCT | 52 | Aerobic exercise | 3x/wk for 60 min; supervised | Intensity set to 50-70% VO2 max at baseline, which was reassessed at 6mo to adjust training intensity | Dietary lessons 1x/wk with set energy intake based on low levels of activity of same age/sex individuals following balance diet  Weekly psychological support group |
|  |  |  | Aerobic + resistance exercise | 3x/wk for 60 min; supervised | 30 min aerobic training with intensity set to 50-70% VO2 max along with 30 min resistance training targeting main muscle groups with 3 sets of 6-20 reps |  |
| De Lira et al. 2017 | RCT | 12 | High intensity training | 3x/wk; supervised | Ventilatory threshold 1; goal 350 kcal expenditure per session; duration depended on reaching expenditure goal | Nutritional counseling 1 hr/wk: focused on education, healthy eating, and balanced diet. No specific prescription for energy intake recommended  Psychological counseling support group 1 hr/wk |
|  |  |  | Low intensity training | 3x/wk; supervised | 20% below ventilatory threshold 1; goal 350 kcal expenditure per session; duration depended on reaching expenditure goal |  |
|  |  |  | Control | N/A | No intervention | No intervention |

Abbreviations: RCT, randomized controlled trial; wk, week; min, minute; VO2 peak, rate of oxygen consumption at peak exercise; reps, repetitions; max, maximum; NRCT, non-randomized controlled trial; m, meter; kcal, kilocalorie; s, second; hr, hour; HRmax, maximum heart rate; N/A, not applicable; VO2 max, maximal rate of oxygen consumption; g, grams.
