# Supplementary material for: Systematic review of exercise for the treatment of pediatric metabolic dysfunction-associated steatotic liver disease
Source: PLoS One. 2024 Dec 10;19(12):e0314542. doi: 10.1371/journal.pone.0314542 (PMC11630624; doi:10.1371/journal.pone.0314542)
Supplement: Attachment — Submitted filename: Response to Reviewers.docx [file pone.0314542.s010.docx]

**Point by Point Response**

**Reviewer #1**

In this systematic review, Smith and colleagues have collected and summarized controlled and non-controlled exercise studies for the treatment of MASLD/NAFLD. Strengths include a rigorous application of the systematic review process and clear presentation of the results. Their findings point towards the lack of high-quality studies in this field, which is important in its own right.
1. One interesting results is that most studies in this field did not require patients to have been diagnosed with NAFLD, thereby introducing heterogeneity within the groups, and lowering the chance of finding significant results. The authors compare the patient characteristics to published cohorts of pediatric NAFLD patients. While this reinforces this point, this step is a bit superfluous in my opinion. Especially comparing serum ALT levels is not straightforward, as biopsy-proven pediatric cohorts themselves are subject to sampling bias (at the other end of the spectrum), as reflected in quite high ALT levels in some cohorts. If the authors choose to retain this comparison, this caveat should be clearly mentioned.

**Response:** We appreciate the reviewer’s insightful comments. The utility of evaluating any given intervention for MASLD/NAFLD is deeply tied to the relevance of the patient population studied. Given the substantial resources required for clinical trials—both in terms of cost and participant commitment—it is essential that these studies focus on the appropriate populations. Otherwise, their potential to inform clinical practice is diminished. We believe it is crucial for the field to recognize this issue, particularly since many studies have not adequately focused on the relevant populations. Therefore, we consider the inclusion of this comparison necessary rather than superfluous.

We included information on clinical trials involving other interventions to highlight the discrepancy between the clinical populations most in need of treatment and those typically enrolled in exercise studies. We agree that patients with MASLD diagnosed in specialty clinics often have more severe disease, which underscores their need for effective treatments. We have added further discussion in the section titled “Gaps in study inclusion” (lines 587-592) to clarify our rationale for this comparison and to address the elevated ALT levels observed in biopsy-proven pediatric cohorts.

Minor points:
2. The most common risk of bias was ‘deviation from the intended interventions’. The reader would be well served if the specifics of this could be discussed in a bit more detail.

**Response:** We agree that a more detailed discussion on the high risk of bias due to ‘deviation from the intended interventions’ would benefit the reader. We have expanded our commentary on this point in the results section, under ‘Summary of risk of bias assessment’ (lines 299-305), to explain how specific studies deviated from their intended interventions.

3. Every study is discussed separately, without presenting a broader view in the results section. I would for instance discuss the two papers by Lee S et al. together.

**Response:** We have presented a broader view of the results in the first paragraph of the discussion (lines 551-564). In response to the reviewer’s suggestion, we have rearranged the results section so that the two paragraphs discussing the studies by Lee S et al. are now presented sequentially. However, we believe that discussing these studies separately is important for clarity and consistency with the treatment of other studies in the review.

**Reviewer #2**

1. I recommend major revision.

**Response:** We appreciate the reviewer’s recommendation for major revisions. In response to the reviewers and editors, we updated the search and revised the tables, figures, and text accordingly.

2. Meta-regression analysis for confounding variables such as age, gender and past medical history.

**Response:** The studies included in this review were not suitable for meta-analysis due to the limited data available for each outcome, significant heterogeneity among the included studies in terms of design, population, intervention, and outcome measures, and the high risk of bias present in 10 out of 16 studies. As a result, a meta-analysis was not conducted. We have added a more detailed explanation in the methods section, ‘Data extraction and management’ (lines 198-201), to clarify this decision. We have also cited a relevant methods paper to support our approach. We hope this systematic review will guide future studies on exercise as an intervention for pediatric MASLD, helping them meet the standards necessary for meta-analysis.

3. There are so many factors that can affect the result, especially the history of exercise. These should be mentioned in the paper.

**Response:** We revisited all the included studies to address the concern regarding exercise history. Unfortunately, we found that this variable was typically not reported and was not evaluated as a covariate in the outcomes of any of the studies.

4. Also, the search strategy must be updated. It is for one year ago.

**Response:** In response to your request, we re-ran the original search strategy on August 8, 2024, to ensure the review’s currency. We also included the more recent terms “MASLD” and “metabolic dysfunction-associated steatotic liver disease.” This search identified one additional study that met the inclusion criteria, which has been incorporated into the systematic review. We have updated the PRISMA flow diagram and the relevant sections of the manuscript accordingly.
